# Unravelling developmental disregard in children with unilateral cerebral palsy by measuring event-related potentials during a simple and complex task

**DOI:** 10.1186/1471-2377-14-6

**Published:** 2014-01-08

**Authors:** Ingar M Zielinski, Marijtje LA Jongsma, C Marjolein Baas, Pauline BM Aarts, Bert Steenbergen

**Affiliations:** 1 Behavioural Science Institute, Radboud University Nijmegen, PO Box 9104, 6500, HE Nijmegen, The Netherlands; 2 Department of Pediatric Rehabilitation, Sint Maartenskliniek, Postbus 9011, 6500 GM Nijmegen, The Netherlands; 3 School of Psychology, Australian Catholic University, 115 Victoria Pde, Melbourne, VIC 3450, Australia

**Keywords:** Unilateral cerebral palsy, Developmental disregard, EEG, Event-related potentials, Cognitive load

## Abstract

**Background:**

In a subset of children with unilateral Cerebral Palsy (CP) a discrepancy between capacity and performance of the affected upper limb can be observed. This discrepancy is known as Developmental Disregard (DD). Though the phenomenon of DD has been well documented, its underlying cause is still under debate. DD has originally been explained based on principles of operant conditioning. Alternatively, it has been proposed that DD results from a diminished automaticity of movements, resulting in an increased cognitive load when using the affected hand. To investigate the amount of involved cognitive load we studied Event-Related Potentials (ERPs) preceding task-related motor responses during a single-hand capacity and a dual-hand performance task. It was hypothesised that children with DD show alterations related to long-latency ERP components when selecting a response with the affected upper limb, reflecting increased cognitive load in order to generate an adequate response and especially so within the dual-hand task.

**Methods:**

Fifteen children with unilateral CP participated in the study. One of the participants was excluded due to major visual impairments. Seven of the remaining participants displayed DD. The other seven children served as a control group. All participants performed two versions of a cue-target paradigm, a single-hand capacity and a dual-hand performance task. The ERP components linked to target presentation were inspected: the mid-latency P2 component and the consecutive long-latency N2b component.

**Results:**

In the dual-hand performance task children with DD showed an enhancement in mean amplitude of the long-latency N2b component when selecting a response with their affected hand. No differences were found regarding the amplitude of the mid-latency P2 component. No differences were observed regarding the single-hand capacity task. The control group did not display any differences in ERPs linked to target evaluation processes between both hands.

**Conclusion:**

These electrophysiological findings show that DD is associated with increased cognitive load when movements are prepared with the affected hand during a dual-hand performance task. These findings confirm behavioural observations, advance our insights on the neural substrate of DD and have implications for therapy.

## Background

Cerebral Palsy (CP) is defined as a group of non-progressive disorders related to the development of movement and posture, caused by damage to the developing foetal or infant brain [1]. A large group (between 21 and 40%) of children with CP is formed by those with unilateral CP, having substantially greater motor deficits in one upper extremity than the other [2]. The observed unilateral motor impairments are related to damage of brain regions involved in planning, controlling, and execution of movements leading to a reduced movement capacity in children with unilateral CP [3]. Apart from the reduced movement capacity, a subset of children with unilateral CP also seem to disregard the preserved capacity of their affected upper limb, leading to a failure to use the affected arm and hand according to its full capacity in daily life [3-6]. This discrepancy between capacity and performance is defined as Developmental Disregard (DD) [3-5].

To date, different explanations have been put forward to explain DD in children with unilateral CP. A common explanation is based on the theory of operant conditioning [7,8]. It compares DD to the phenomenon of learned non-use, defined as a learned suppression of movement, reported in the literature in adults who suffered a cerebrovascular accident (CVA) [9]. Following this theory, it is suggested that children with DD have experienced too little incentive to use the affected upper limb, because using the unaffected limb is less demanding [5]. Thus, positive reinforcement resulting from the successful use of the unaffected upper limb is combined with negative reinforcement from the unsuccessful use of the affected upper limb. This leads to a behavioural bias favouring the unaffected limb disproportional to the capacities of both the unaffected and affected upper limb.

Despite the similarity of the behavioural symptoms associated with learned non-use in CVA patients and DD in children with unilateral CP, recent studies emphasize that in DD both the developmental aspect and related cognitive aspects of information processing pose an important conceptual difference to the pure behavioural phenomenon described in learned non-use [3-6,10,11]. In this respect, Deluca and colleagues [5] have postulated that children with DD have suffered a critical lack of movement stimulation during developmental periods when movement repertoires are rapidly acquired in typically developing children. As a consequence of this lack of movement that starts at perinatal periods, in combination with the earlier mentioned effects of reinforcement, typical developmental milestones are delayed or even deficient for the affected upper limb. In line, the neural substrates involved in motor control as well as in sensori-motor integration of the affected limb experience a similar lack in development and refinement [5,10,12]. It has even been stated that DD might be a neurologically based phenomenon similar to poststroke neglect syndrome [12].

In a recent explanation to account for DD this protracted development of motor control, sensori-motor integration and linked neural substrates is suggested to cause certain movement patterns of the affected arm and hand to be not sufficiently automated [4]. Based on Fitts and Posner’s [13] theory of motor skill acquisition, Houwink and colleagues [4] hypothesised that due to the lack of automaticity, using the affected upper limb requires a disproportional amount of attention [4]. They argue that a disproportional amount of attention coincides with an excess in cognitive load that is associated with motor control of the affected upper limb. The increased cognitive load in turn leads to a reduced spontaneous use of the affected arm and hand in daily life [4]. This hypothesis was already verified in several studies with CVA patients. These studies showed that patients, who have to relearn a lost motor skill, need a disproportional level of attention when moving the affected limb in the early stages of rehabilitation when relearned movements are not yet (re)automated [14,15]. Thus, a lack of automaticity of movements is associated with increased cognitive load in adult CVA patients.

To be able to assess cognitive load related to movement, Event-Related Brain Potentials (ERPs) offer the unique opportunity to directly measure neural responses associated with distinct processing stages preceding an overt response [16]. Whereas mid-latency components (e.g. N1 & P2) have been associated with orienting and perception, the long-latency components of ERPs (e.g. N2, P3) are known to reflect processes associated with cognitive control and attention allocation [17,18]. To assess the possible role of cognitive load in the impaired motor performance of DD, the current study therefore focussed on the long-latency N2b component. Next to generally being known to reflect processes associated with cognitive control and attention allocation, the N2b has also already directly been linked to cognitive control of response-related processes [19].

In order to investigate the aspects of information processing preceding goal directed motor responses, ERPs were extracted from the ongoing EEG during a single-hand task as an index of the individuals hand capacity and a dual-hand task, to estimate the hand performance. Based on the cognitive load theory of DD we reasoned that children with DD will show alterations linked to the higher order cognitive control processes when preparing a response with their affected upper limb during the dual-hand performance task. We therefore hypothesize that children with DD show alterations related to the N2b component when selecting a response with the affected upper limb, reflecting increased cognitive load in order to generate an adequate response. We furthermore hypothesize this effect to be especially pronounced during the more demanding dual-hand performance task.

## Methods

### Participants

Fifteen children diagnosed with unilateral Cerebral Palsy (CP; 5 girls, 10 boys, *M*_*age*_ *=* 8 years, 1 month, age range: 5 years, 3.5 months - 11 years, 1.5 months) were recruited from the Sint Maartenskliniek, Nijmegen, the Netherlands. One participant was excluded form the final analyses due to major visual impairments (diagnosed with hemianopsia), which may have confounded our results. Side of affected hand, manual ability, as well as Developmental Disregard (DD), of each individual child was assessed by an occupational therapist prior to the EEG measurements. Manual ability of each child was assessed using the Manual Ability Classification System (MACS) for children with CP [20]. Groups were classified using the “Video-Observation Aarts and Aarts module: Determine Developmental Disregard” (VOAA-DDD-R) [21].

Seven children were classified as having DD (*M*_*VOAA-DDD-R*_ = 21.4, SD_*VOAA-DDD-R*_ *= 6.7; M*_*age*_ *=* 7 years, 9 months, *SD*_*age*_ = 1 year, 11 months; 6 male, 1 female; left vs. right hand affected: 5/ 2; *M*_*MACS*_ = 1.6, *SD*_*MAC*S_ = 0.5). The other seven children served as the control group, that is, children with unilateral CP but without DD (*M*_*VOAA-DDD-R*_ = 4.8, SD_*VOAA-DDD-R*_*=*10.6 *; M*_*age*_ *=* 8 years, 11 months, *SD*_*age*_ = 2 years, 1 month; 3 male, 4 female; left vs. right hand affected: 5/ 2; *M*_*MACS*_ = 1.9, *SD*_*MAC*S_ = 0.7 ).

To test whether the groups did not differ with respect to age, gender, side of the affected limb, and manual ability (MACS), independent-samples Mann-Whitney U Test were conducted. No differences were observed for either of these variables.

Approval for the experiment was obtained from the local Ethical Committee of the Faculty of Social Sciences (EC), Radboud University Nijmegen (Registration number: 2012/049; NL nr.: 39607.091.12). The parents of all participants signed a written informed consent form prior to the study for their children to participate in the study and for the participant information to be used for research purposes.

### Design

In this experiment two versions of a cue-target paradigm were used. Cue and target stimuli were embedded within a train of background stimuli. The stimuli were sequentially presented in a semi-random order so that every cue stimulus was followed by a target stimulus but the occurrence of the cue stimulus within the train of background stimuli was random. The probability of the occurrence of both the cue and target stimuli was 0.25 (half of the stimuli were background stimuli).

**Figure 1:**
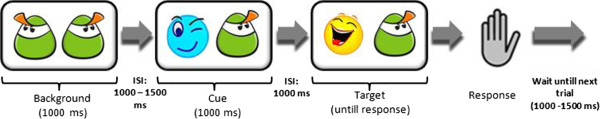
**Stimuli of cue-target paradigm.** Schematic of the cue-target paradigm shown on the left side of the screen. For presentation on the right side, the smiley figures were mirrored horizontally.

All stimuli consisted of a pair of “smiley” figures: one on the left side of the screen and one on the right side of the screen. Cue stimuli consisted of a blue (cue) smiley figure paired with a green (background) smiley figure. Target stimuli consisted of a yellow (target) smiley figure paired with a green (background) smiley. The target was always presented at the same side as the preceding cue. Background stimuli consisted of two paired green smiley figures. Smiley figures (size 7x7 cm) were presented at a fixed position with a white background on a laptop screen approximately 40 cm in front of the child. Figure 1 provides a visual presentation of the stimuli.

The stimulus duration of background- and cue-stimuli was 1000 ms. Target stimuli were presented until the child responded. The inter-stimulus interval (ISI) between cue and target stimuli was kept fixed at 1000 ms. The ISI after background stimuli and after responses was set randomly between 1000 and 1500 ms. Participants were instructed to respond to target stimuli by pressing a button at the same side at which the target was presented (right or left) as quickly as possible with the corresponding hand. For this purpose two red buttons (diameter: 9.5 cm; height: 5.5 cm) were located next to the laptop keyboard, one at the right side and one at the left side. The distance between these buttons was kept at 30 cm to prevent that the wrong hand was used to press the according button. Only after a response was recorded (correct or incorrect) the next trial was started. After each correct response a short laughing sound was presented to provide feedback. No sound was presented after incorrect responses. Incorrect responses were defined as erroneous responses to cue or background stimuli, incorrect responses following target stimuli (using the wrong hand), as well as omissions following target stimuli (no response within 2000 ms). The experiment was divided into two blocks related to the two different tasks (single-hand capacity vs. dual-hand performance task). In the first block the trains of cue and target stimuli were only presented at one side of the screen (single-hand task), starting with the side corresponding to the non-affected upper limb and followed by the side corresponding to the affected upper limb. Each run contained 25 trains of cue-target stimuli (a total of 50 target stimuli, 25 for both sides). In the second block, the cue and target stimuli were shown in a semi-random order at either the left or right side of the screen (dual-hand task), demanding alternating responses of either the right or left hand. Twenty trains of cue-target stimuli were presented on the left side and 20 trains of cue-target stimuli at the right side of the screen (a total of 40 target stimuli).

### EEG recordings

EEG signals were recorded with a 32-channel actiCap (MedCaT B.V., the Netherlands) and subsequently amplified by a 32-channel BrainAmp EEG amplifier with electrode placement according to the International 10-20 system [22,23]. A ground electrode was placed over AFz and a reference over the left mastoid bone. The EEG signal was offline re-referenced to linked mastoids and stored on disk for offline analyses. Vertical and horizontal eye movements were recorded by two additional bipolar channels placed above and below the right eye and on the outer canthi of each eye. Electrode impedance was kept below 5 kΩ. The signal was digitized at 1000 Hz and filtered online between 0.016 Hz (i.e. 10s time-constant) and 250 Hz. Electrodes were located at five midline sites (Fz, FCz, Cz, Pz and Oz) and 24 lateral sites (FP1/2, F7/8, F3/4, FC5/6, FC1/2, C3/4, CP5/6, CP1/2, P7/8, P3/4, T7/8, O1/2) to allow estimations of scalp distributions for finding spatial maxima of the ERP components of interest.

### Procedure

Prior to the EEG measurements children were assessed by a clinician to be tested for side of affected hand and DD. Next, the EEG and Electrooculography (EOG) electrodes were placed (approximately 30 to 45 minutes) and the child was seated in front of the laptop screen on a comfortable chair adjusted to the correct height. Recordings were done at a familiar setting (Rehabilitation Centre Sint Maartenskliniek, Nijmegen, the Netherlands). At least one of the parents was always present during recordings.

The child received instructions before each block of the experiment by showing the stimuli and pointing out which button to press. A short practice session preceded each block to familiarize the child with the task. The whole procedure did not exceed 90 minutes.

### Data processing and analysis

EEG data were analysed using the software BrainVision Analyzer v. 2.0 (Brain Products GmbH). For each participant an ocular correction was applied using a semi-automatic correction procedure based on the logarithm of Gratton and Coles [24]. Next, the EEG signal was high-pass filtered at 0.5 Hz and low-pass filtered at 30 Hz. Based on the onset of all cue and target stimuli the EEG was segmented into epochs from -250 ms pre-stimulus to 750 ms post-stimulus. Only ERPs corresponding to correct responses following cue (no response) and target stimuli (response within 2000 ms) were included using the Advanced Boolean Expression (total of 93.04% of the trials). After segmentation epochs were de-trended and artefacts related to gross motor movement and muscle tension were removed manually. Next, a baseline correction (-250 – 0 ms) was applied to all segments.

All segments were averaged per stimulus type (cue vs. target), hand (affected vs. non-affected), and task (single-hand vs. dual-hand). ERP components were defined in terms of their polarity, latency, and scalp distribution. The grand average ERPs following both cue and target stimuli contained a clear N1 (mean latency: 130 ms), P2 (mean latency: 215 ms), and N2b component (mean latency 355 ms) component. Based on conventionally reported and observed scalp distributions of the N1, P2, and N2b, component amplitudes at FCz were further analysed [25-27]. To allow blind scoring, ERP amplitudes were defined as the averaged value within a fixed latency window: N1 (120 – 140), P2 (200 – 230 ms), and N2b (330 – 380 ms) [28]. ERP mean amplitude of the cue and target ERP components were analyzed separately using repeated measures GLM analyses with handedness (affected vs. non-affected hand) and task (single-hand vs. dual-hand task) as independent within-subject variables and group (control group vs. DD group) as between-subject factor. Whenever interaction effects were observed appropriate Paired-Samples T-Tests were performed. For all analyses the significance level was set at α < .05.

Analysis of behavioural responses focused on Inverse Efficiency Scores (IES) determined as the mean reaction time (RT) divided by the proportion of correct responses expressed in ms [29]. This method is considered to be especially useful in tasks with low (<10%) error rates (Bruyer & Brysbaert, 2011). Indeed, error rates of the current experiment remained below 7% for the whole group. IES scores were analyzed using repeated measures GLM analyses with handedness (affected vs. non-affected hand) and task (single-hand vs. dual-hand task) as independent within-subject variables and group (control group vs. DD group) as between-subject factor. For all analyses the significance level was set at α < .05.

## Results

To test our first hypothesis that children with DD compared to children with unilateral CP without DD show alterations related to long-latency ERP component when selecting a response with the affected upper limb compared to the non-affected upper limb, repeated measures GLM analyses of the long-latency N2b ERP component were performed. To ensure that differences can not be ascribed to earlier processes related to the evaluation of the physical features of task relevant stimuli, the mid-latency N1 and P2 components were also inspected. To furthermore ensure that differences could also not be ascribed to early cue evaluation or general visual stimulus evaluation processes, ERP components following cue stimuli were investigated as well.

The repeated measures GLM analyses of the ERP components following cue stimuli revealed no significant interaction or main effects with respect to the N1 component (all *p’s* > .10), the P2 component (all *p’s* > .10), or the N2b component (all *p’s* > .10). The analyses of the ERP components following target evaluation revealed no significant interaction or main effects with respect to the mid-latency N1 (all *p’s* > .10) and P2 components (all *p’s* > .10). With respect to the long-latency N2b component this analysis did however reveal a significant task (single vs. dual task; *F*(1,13) = 5.265, *p* = .041, η_p_^2^ = .288) effect as well as a significant handedness x task x group interaction (*F*(1, 13) = 6.649, *p* = .026, η_p_^2^ = .338).

**Figure 2:**
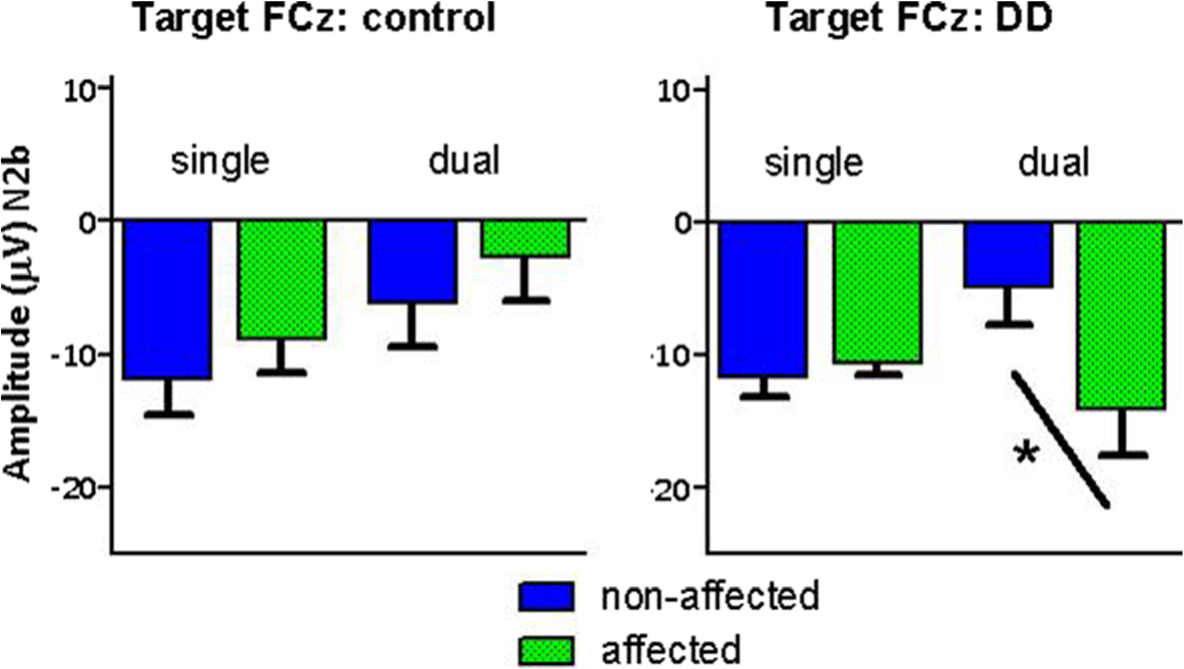
**Mean absolute amplitude with SEM for the N2b (330 - 380) component following target evaluation at FCz.** Differences between sides of target presentation (NA = non-affected; A = affected) are depicted for children with unilateral CP without indications of DD (control) and children with unilateral CP and DD (DD). The significant difference between the movement preparations of both hands for the DD group is indicated by the asterisk.

**Figure 3:**
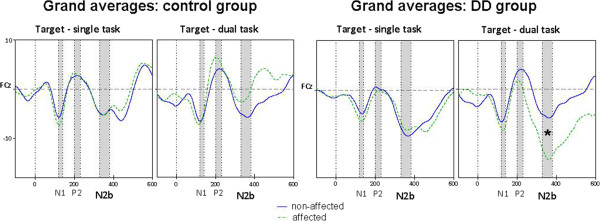
**Grand averaged ERP waveforms following target stimuli.** Grand averaged ERP waveforms elicited by target stimuli in children with unilateral CP without indications of DD (Grand Averages: control group) and children with unilateral CP and DD (Grand averages: DD group) in response to movement selection of the affected hand (dashed line) compared to the non-affected hand (solid line). For calculation of these grand averages all 14 participants (DD: N = 7; control: N = 7) were included. Highlighted temporal windows indicate N1, P2, and N2b components. The significant difference for the DD group between the movement preparations of both hands is indicated by the asterisk.

To examine this interaction and to test our second hypothesis, that the long-latency effect is especially pronounced during the more demanding dual-hand performance task paired sample t-tests between hands (affected vs. non- affected hand) were performed for each group and task separately. This revealed that the significant difference between hands was only present in the DD group and only in the dual-hand task (t(6) = 2.469, *p* < .05). Specifically, only in the dual-hand task the N2b amplitude following target stimuli was significantly enhanced in the DD group when using the affected hand compared to using the non-affected hand, confirming our first and second hypothesis. Grand average ERPs to target stimuli for both the DD and the control group are depicted in Figure 2. provides a visual presentation of the mean absolute amplitude for the N2b component following target stimuli. No differences between hands were observed in the control group Figure 3.

Finally, with respect to the behavioural data the repeated measures GLM analyses of the IES scores revealed a significant main effect of hand (*F*(1, 13) = 5.715, *p* = .033, η_p_^2^ = .305). Across both group responses were less efficient with the affected hand (*M* = 598.17, *SD* = 256.48) compared to responses with the non-affected hand (*M* = 558.38, *SD* = 227.28). No GLM interaction effects with respect to the IES scores were observed. The IES scores were similar for both groups.

## Discussion

The goal of the current study was to use Event-Related Potentials (ERPs) to provide a direct measure of cognitive load associated with movements of the affected upper limb in children with unilateral Cerebral Palsy (CP) and Developmental Disregard (DD). Based on Fitts and Posner’s [13] theory of motor skill acquisition, it has been suggested that due to a lack of automaticity of movements with the affected upper limb, children with DD experience increased cognitive load when using this limb [4]. This increased cognitive load in turn leads to an underuse of the affected arm and hand in daily life performance even if sufficient limb capacity is available. In order to test this theory we recorded ERPs during two different versions of a cue-target paradigm. First, we employed a single-hand task as an index of the individuals hand capacity. Next, we recorded a dual-hand task to estimate the hand performance. We first of all hypothesized that children with DD would show alterations related to the long-latency ERP components when selecting a response with the affected upper limb, reflecting increased cognitive load when generating an adequate response. Secondly, we hypothesised this effect to be especially pronounced during the dual-hand performance condition, reflecting the characteristic discrepancy between hand capacity and hand performance of DD.

In line with our first hypothesis children with DD showed an enhancement in mean amplitude of the long-latency N2b ERP component when preparing a response with their affected compared to their non-affected hand. This component is well known to reflect the amount of activity in areas associated with cognitive load, indexes voluntary attentional processing, and is known to represent a necessary part of the information processing sequence leading to a motor response [30,31]. The finding of the increased N2b following target stimuli in children with DD therefore indicates that these children experience an increase in cognitive load when generating an adequate motor response with their affected compared to their non-affected hand. Moreover, and in line with our second hypothesis, this enhancement was only observed in the dual-hand performance task and not in the single-hand capacity task.

Based on our electrophysiological results the conclusion is warranted that the discrepancy between capacity and performance in children with DD can be explained by an increased cognitive load associated with response selection in dual-hand task situations. This conclusion is further strengthened by three findings of the present study. First, the enhancement of the N2b ERP component following target stimuli when generating a response with the affected hand was not found in the control group. Second, we did not find any differences between hands with respect to the mid-latency N1 and P2 amplitude. This indicates that there are no differences between both sides of target presentation regarding the evaluation of the physical features of task relevant stimuli [32,33]. Observed group differences can therefore not be explained due to differences in processes related to orienting and perception that regularly accompanies CP [34]. Third, and finally, there were no differences regarding cue evaluation processes preceding the target stimuli. This finding shows that visual and cognitive evaluation processes that are not directly linked to preparing a motor response following target evaluation are not impaired in children with DD compared to children with unilateral CP without DD.

The fact that preparing a response with the affected upper limb compared to preparing a response with the non-affected arm and hand increases cognitive load in a dual-hand performance task is in line with the behavioural observation of the discrepancy between capacity and performance that characterizes DD [4]. The current study showed that children with DD use their affected hand during a single-hand capacity task without any electrophysiological or behavioural indications of increased cognitive load when preparing the response. This finding is in agreement with the behavioural observation that children with DD are indeed able to perform a particular task with their affected hand alone, as long as they can focus on the task and their hand capacity is sufficient [4,10]. However, we did observe an increased ERP N2b component when preparing a response with the affected upper limb in a dual-hand performance task in children with DD reflecting increased cognitive load. This finding exemplifies the behavioural observation that in spontaneous daily use, predominantly requiring both hands, children with DD fail to use the potential motor functions of their affected limb and rather chose to perform a task with their non-affected upper limb alone [4,10]. In this connection, an interesting facet of the current study regarding the behavioural observations of DD is that there were no differences between groups regarding the behavioural efficiency scores. That is, even though our electrophysiological findings indicate increased cognitive load associated with the use of the affected upper limb in a dual-hand task an appropriate movement outcome could be achieved. These findings further substantiate the claim that DD is due to increased cognitive load associated with the use of the affected upper limb that only reveals itself in complex activities where attention cannot be solely focused on the effected arm and hand. Children with DD are able to use the affected hand efficiently even in dual-hand tasks but due to the enhancement of cognitive load associated with this movements they disregard their hand in spontaneous daily live.

In sum, the results of the current study add to the accumulating evidence suggesting that cognitive aspects of information processing play a major role in the appearance of DD [3-6,10,11]. Furthermore, these results are in line with the assumption that DD might be a neurologically based phenomenon similar to poststroke neglect syndrom [12]. It is already known that motor neglect becomes worse when attention is distracted and that simultaneous movement of the opposite limbs may also increase motor neglect [35]. This comparison would also be in line with the theory that due to an asymmetrical development of the affected limb, new neural substrates for entire classes of behavior are not well established, refined, and coordinated [5,10,12].

Next to making a substantial step in unravelling DD in children with unilateral CP, these results have important clinical implications. To date, a very commonly applied therapy aimed at improving the upper limb capacity in all children with unilateral CP is the so called ‘forced-use’ or ‘Constraint Induced Movement Therapy’ (CIMT) [36]. The main characteristic of this therapy is the immobilization of the non-affected upper limb, thus forcing the patient to use the affected limb exclusively [36]. By applying this therapy the capacity of the effected arm and hand is intensively trained and often improves spectacularly [37-40]. However, CIMT was originally developed to overcome learned non-use in adult CVA patients and to promote use of the limb rather than skill [36]. In children, however, compared to adult patients developmental factors play a major role in the occurrence of DD [3-6,10,11] whereby they may have never learned how to effectively use their more affected upper limb during many tasks [6,41]. Gordon [41] therefore concluded that treatments for children to overcome DD must be developmentally focused and must take into account the importance of motor learning. This critical view on applying CIMT to children with DD is strengthened by the findings of the current study. We showed that the performance issues typically observed in DD are directly related to increased cognitive load only when using the affected hand in a dual-hand performance task and not when simply moving that limb in a single-hand task. It should therefore be considered to apply bimanual training instead of CIMT to children with DD to promote bimanual skill instead of unimanual use.

Recently, CIMT treatments have been combined with, or compared to, bimanual training therapies [40-45]. These studies show that the results of bimanual training therapies as well as a combination with CIMT are very beneficial and lead to similar improvements in hand capacity as CIMT. Furthermore, they showed that bimanual training leads to a further improvement in bimanual skill and self-determined goals.

Next to the promising consideration to apply bimanual training therapies to children with DD it should also be considered that upper-limb training should not end after an intensive rehabilitation program, but to be continued and integrated in the daily-live activities of the children with DD [4]. As been shown in adult CVA patients increased cognitive load is directly associated with a lack of automaticity of using the affected upper limb [14,15]. In the current study we demonstrated that during hand movements of the affected hand in a dual-hand performance task a disproportional amount of cognitive load is activated suggesting a lack of automaticity of using that hand only during dual-hand performance tasks. Continuing and integrating the rehabilitation program in the daily-live activities of the children with DD therefore has to be considered to promote further automatisation of movements of the affected hand during daily dual-hand performance. In this respect it has already been reported that concerning the daily performance of the affected arm and hand in children with unilateral CP treatment is more effective when conducted in the home setting of the children compared to the clinical setting [46]. In the study of Rostami and Malamiri [46] it was concluded that this natural daily-life environment provides more information about upper limb performance than other contexts such as the clinic. Considering continuing training in a home setting after completing a bimanual rehabilitation program is therefore a crucial next step in reducing DD.

Study limitations include small sample size and therefore difficulties controlling for any interaction between gender and maturation. A further limitation of the current study, also related to the small sample size, is the heterogeneity of the studied group. This latter limitation is however inherent to the participant population as unilateral Cerebral Palsy comprises a very heterogeneous group of movement disorders.

## Conclusions

The discrepancy between capacity and performance in children with DD can be explained by an increased cognitive load associated with response selection in dual-hand task situations. The results of the current study therefore provide direct neurophysiological evidence to the accumulating indications suggesting that cognitive aspects of information processing play a major role in the appearance of DD. Furthermore, by showing that the performance issues typically observed in DD are directly related to increased cognitive load only when using the affected hand in a dual-hand performance task and not when simply moving that limb in a single-hand task it can be concluded that bimanual training, instead of CIMT, should be applied as therapy to children with DD.

## Abbreviations

CIMT: Constraint induced movement therapy; CP: Cerebral palsy; CVA: Cerebrovasculair accident; DD: Developmental disregard; EEG: Electroencephalography; EOG: Electrooculography; ERP: Event-related potential; GLM: Generalized linear model; IES: Inverse efficiency scores; RT: Reaction time.

## Competing interests

The authors report no conflicts of interest.

## Authors’ contributions

All authors contributed equally to the study. All authors have read and given final approval of the final version of the manuscript to be published.

## Pre-publication history

The pre-publication history for this paper can be accessed here:

http://www.biomedcentral.com/1471-2377/14/6/prepub
